# Recovering from a bad start: rapid adaptation and tradeoffs to growth below a threshold density

**DOI:** 10.1186/1471-2148-12-109

**Published:** 2012-07-04

**Authors:** Christopher J Marx

**Affiliations:** 1 Department of Organismic and Evolutionary Biology, Harvard University, Cambridge, MA 02138, USA; 2 Faculty of Arts and Sciences Center for Systems Biology, Harvard University, Cambridge, MA 02138, USA

## Abstract

**Background:**

Bacterial growth in well-mixed culture is often assumed to be an autonomous process only depending upon the external conditions under control of the investigator. However, increasingly there is awareness that interactions between cells in culture can lead to surprising phenomena such as density-dependence in the initiation of growth.

**Results:**

Here I report the unexpected discovery of a density threshold for growth of a strain of *Methylobacterium extorquens* AM1 used to inoculate eight replicate populations that were evolved in methanol. Six of these populations failed to grow to the expected full density during the first couple transfers. Remarkably, the final cell number of six populations crashed to levels 60- to 400-fold smaller than their cohorts. Five of these populations recovered to full density soon after, but one population remained an order of magnitude smaller for over one hundred generations. These variable dynamics appeared to be due to a density threshold for growth that was specific to both this particular ancestral strain and to growth on methanol. When tested at full density, this population had become less fit than its ancestor. Simply increasing the initial dilution 16-fold reversed this result, revealing that this population had more than a 3-fold advantage when tested at this lower density. As this population evolved and ultimately recovered to the same final density range as the other populations this low-density advantage waned.

**Conclusions:**

These results demonstrate surprisingly strong tradeoffs during adaptation to growth at low absolute densities that manifest over just a 16-fold change in density. Capturing laboratory examples of transitions to and from growth at low density may help us understand the physiological and evolutionary forces that have led to the unusual properties of natural bacteria that have specialized to low-density environments such as the open ocean.

## Background

“For me, encountering the bacterial growth curve was a transforming experience. As my partner and I took samples of the culture at intervals to measure optical density and plotted the results on semilogarithmic paper, we saw, after the lag period, a straight line developing. . .beautiful in precision and remarkable in speed. As the line extended itself straight-edge true, I imagined what was happening in the flask—living protoplasm being made from glucose and salts… I had just witnessed the working out of the mathematical statement of that property, dN/dt = kN (where *N* is the number of cells or any extensive property thereof, *t* is time, and *k* is the first-order rate constant [in reciprocal time units]).”

The above quote from a Guest Commentary written by Frederick Neidhardt [1] captures beautifully the remarkable property of exponential growth. As Monod and colleagues had worked out, one can think of the maximum growth rates and substrate affinities of entire cells in precisely the same framework as Michaelis-Menten kinetics describe the reaction rates of individual enzymes. Despite myriad simultaneous processes occurring within the cells themselves, the resulting growth is beautifully and remarkably consistent. Upon these grounds it has become possible to measure growth attributes of single genotypes and quantitatively predict the outcome of competitions, whether or not they occurred in the constant, single nutrient-limited worlds of chemostats [2,3] or the changing, seasonal world of batch cultures [4]. An implicit assumption of the majority of such work, and indeed much of microbiology, is the individualistic nature of planktonic bacteria. Under this assumption, the growth of a given genotype is solely determined by the properties of the external environment provided by the researcher and the intrinsic capacities of the cell. Besides competition for substrates, cell growth is autonomous, and they are blissfully unaware of each other.

There are of course known examples that tarnish this simplistic picture of growth as an unwavering exponential process fully captured by an internal capacity for expansion, such as accumulation of toxic products, changes in pH, or declines in dissolved O_2_. These are unsurprising cases where growth of a given cell is negatively affected by the action of other cells in the medium, but what about positive interactions between cells? Even in well-mixed liquid cultures there is a growing list of public goods produced by cells that end up aiding the population as a whole, such as secreting invertase to cleave sucrose [5-7] or the production of secondary metabolites such as metal chelating siderophores or quorum sensing molecules [8,9].

Where there is positive feedback between the interactions between cells and their behavior, as was famously shown for quorum sensing, one typically observes remarkable changes in the population phenotype above threshold densities [10]. In one example, *Micrococcus luteus* grown to stationary phase in lactate minimal medium takes ~100 h to initiate growth if the initial cell density is between 10^1^ – 10^7^ cells mL^-1^, three-fold longer than if started at 10^8^ – 10^9^ cells mL^-1^[10]. Recently, it was shown that solitary (i.e., not aggregated) yeast cells could only grow on sucrose above a critical cell density due to diffusion and loss of the hydrolytic products from invertase [7]. These dynamics around a critical threshold cell density due to feedbacks with cell physiology are not unlike the bistability that occurs within cellular signaling networks with similar feedback, such as the *lac* operon of *Escherichia coli*[11]. Below a critical level of input the *lac* operon remains off, but due to positive feedback from the linkage between induction and increased transport capacity for the inducer, once cells flip on they rapidly become fully induced. This switch-like behavior can allow two different phenotypic states to be maintained in the same environment depending upon the history of the cells, a general phenomenon known as hysteresis. Over half a century ago it was shown that *E. coli* cultures that had previously been grown with a lactose analog (thiomethyl-β-d-galactoside, TMG) remain induced at an intermediate TMG concentration that was incapable of triggering induction in unexposed cells, and that this phenotypic ‘memory’ persisted for 180 generations [12].

Comparative analyses of natural organisms and limited evidence from laboratory evolution both suggest that there are evolutionary tradeoffs between success in low and high densities. Organisms that live at low nutrient concentrations, such as the open ocean, are often ‘oligophiles’. Given that many appear capable of growth only at low substrate concentrations, the use of low nutrient conditions such as C-free minimal medium with agar has been demonstrated to cultivate previously uncultured microbes [13]. Furthermore, many oligophiles halt growth at low densities. For example, the prevalent planktonic bacterium *Pelagibacter ubique* from the SAR11 clade only grows to a final density of ~1 × 10^6^ cells mL^-1^[14]. From an experimental perspective, it has been seen that selection of cultures in a chemostat – where the stoichimetrically limiting resource for growth is held at low concentrations – repeatedly has led to increased expression of transporters for that limiting resource [3,15]. *E. coli* populations grown in lactose-limited chemostats, for example, are rapidly dominated by mutants that render the *lac* operon constitutive and thereby express high levels of the lactose:proton antiporter encoded by *lacY*. Remarkably, this phenotype comes at a tremendous cost at high lactose concentrations: upon plating to agar plates with high lactose the cells die due to rapid, uncontrolled depolarization of proton motive force, a phenomenon termed as “lactose killing” [16]. These genotypes illustrated tradeoffs resulting from a change in nutrient concentration, but I am unaware of a prior example of adaptation to growth at low cell density itself.

The present work involves the fortuitous discovery of a density threshold for growth for a metabolically engineered strain of *Methylobacterium extorquens* AM1. *M. extorquens* AM1 has been the best-studied example of growth on single-carbon (C_1_) compounds due to its genetic tractability and ability to grow on a limited number of multi-C compounds. Previous work established that formaldehyde is generated during growth on substrates such as methanol or methylamine, and is further oxidized to formate via a pathway that uses the C_1_-carrier tetrahydromethanopterin (H_4_MPT) [17,18]. The resulting formate is split: some is fully oxidized to CO_2_, while the remainder is assimilated into biomass via the successive action of a pathway linked to tetrahydrofolate (H_4_F) and the serine cycle [19-22]. The formaldehyde-oxidizing role of the H_4_MPT pathway can be replaced by introducing an alternative system from *Paracoccus denitrificans* that works via C_1_-glutathione (GSH) intermediates [18,23,24]. The resulting strain recovers the ability to grow on C_1_ compounds, but does so three times more slowly than wild-type [25].

Eight replicate populations were evolved to explore how metabolic systems adapt to the introduction of new modules, as can occur naturally due to horizontal gene transfer or by design by metabolic engineers. An engineered *M. extorquens* AM1 strain (hereafter ‘EM’) with the foreign GSH-dependent formaldehyde oxidation pathway in place of the native H_4_MPT-dependent system was evolved in methanol minimal medium (populations ‘F1-F8’) for 900 generations. Key findings from these populations have been the discovery of: 1.) rapid, methanol-specific adaptation due to beneficial mutations occurring in both the introduced pathway and the host genome [25], 2.) a diverse array of mutational types and targets within the plasmid bearing the GSH pathway, all of which optimized the balance between benefits and costs of gene expression [26], and 3.) epistatic interactions between beneficial mutations that co-occurred in one lineage led to the discovery of a generic pattern of diminishing returns that contributes to the deceleration of adaptation [25].

Here I describe that, prior to founding the eight F populations described above, the identical EM ancestor had actually been evolved in the same methanol minimal medium (populations ‘E1-E8’) but behaved quite differently due an unexpected density-dependence for growth. I established that the EM strain – when starting at its typical final density (~1 × 10^8^ mL^-1^) – could comfortably recover to this level over four days following a 64-fold dilution (i.e. six generations). As is standard practice in experimental evolution, when replicate populations were established from the EM ancestor, each flask was inoculated from a unique colony. This ensures that identical mutations in different populations, if observed, can be attributed to independent events (versus possibly arising from parallel selection of a rare, pre-existing rare variant that arose during the ~30 generations required for the outgrowth of the starter population). Because of this, each evolving population was only seeded with some fraction of the cells present in a single colony (which we have previously estimated to be ~ 1 × 10^6^) [27]. At most, this represents a 15-fold lower cell number than the inoculum used in preliminary experiments. As it turned out, when the serial passages began for the eight E populations, the final cell number for six of these transiently plummeted by at least 60- to 400-fold over the first four transfers (which was avoided for the later F populations with a slight change to the first transfer environment). Whereas five of these recovered by 60 generations, one population (E2) remained more than an order of magnitude less dense beyond 120 generations before eventually achieving full density. Sustained culturing below a cell density threshold led to dramatic evolutionary consequences in the E2 population. The fitness of this population – when assayed at ‘standard’ high densities – dropped substantially relative to the EM ancestor. Identical competitive fitness assays at the same ratio of competitors, but simply diluted by an extra 16-fold, demonstrated that the E2 population was actually more than three-fold more fit at this low initial density. Besides serving as a cautionary tale for researchers conducting experimental evolution, future work to uncover the basis of this type of phenomenon in the laboratory may hold relevance for understanding natural microbes that have evolved to the physiological consequences of life at low cell densities.

## Results

### Initiation of replicate populations from single colonies led to variable final densities that persisted for many growth cycles

Eight populations (E1-E8) were established in methanol minimal medium from the EM ancestor that carried a foreign formaldehyde oxidation pathway in place of its native one [25]. Single colonies of the otherwise isogenic pink (CM701) or white (CM702) versions of EM were used to inoculate 9.6 mL of medium. Every four days, 1/64 of the culture was transferred to fresh medium, which allows for an average of 6 generations per cycle. Preliminary experiments established that the EM strains could maintain themselves under this regime if begun at the typical final density of ~1 × 10^8^ mL^-1^, as expected from their growth rate (0.062 ± 0.001 h^-1^; SE reported throughout; doubling time of 11.1 h).

**Figure 1:**
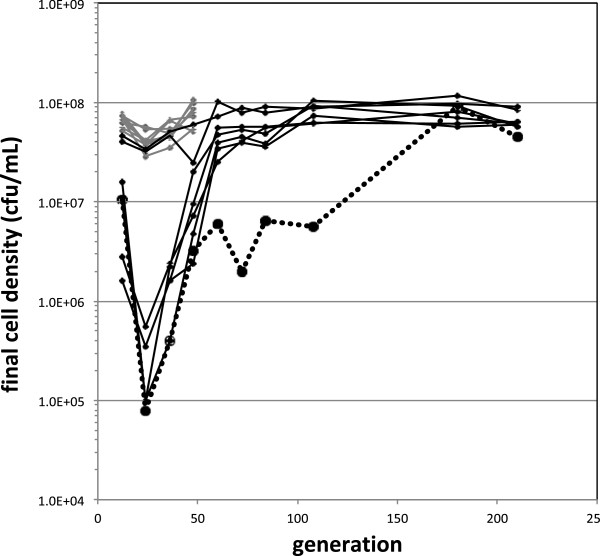
**Transient crash in final cell densities over the course of adaptation.** Colony counts from dilutions of the final population sizes at various transfers indicated that six of eight E populations (shown in black) crashed before recovering. The E2 population (indicated with large circular symbols and a dotted line) stayed at moderately low densities until after 120 generations. In contrast, the F populations (grey, first 48 generations displayed), whose medium was supplemented with the rapidly-consumed substrate succinate for the first growth cycle to assure full density was reached from single colonies, did not exhibit this pattern.

Unexpectedly, most of the populations experienced a severe drop in final density over the first 60 generations (Figure 1). Populations E4 and E5 remained fairly steady, never dipping below 3 × 10^7^ cfu mL^-1^ and achieving a steady return to ~1 × 10^8^ mL^-1^ by generation 72. In contrast, none of the other six populations had a density above 1.6 × 10^7^ mL^-1^ at generation 12, and by generation 24 reached their minimum final cell densities of between 1-5 × 10^5^ mL^-1^. Five of these populations gradually recovered to 2.5-5.5 × 10^7^ mL^-1^ by generation 60, and achieved ~1 × 10^8^ mL^-1^ by generation 108. The remaining population, E2, recovered to only 2-6.4 × 10^6^ mL^-1^ between generation 48 and 120. By 180 generations, even E2 achieved the final density of ~1 × 10^8^ mL^-1^ typical for all lines.

### Populations allowed to recover to full density during the first transfer prevented later declines

Given the unexpected behavior of the E populations, an identical experiment was established in a manner designed to circumvent the problems with insufficient cell density. Although EM grows three-fold worse than wild-type in methanol, it is only ~20% less fit in succinate [25]. For populations F1-F8, individual colonies were used to inoculate each flask as before, but this time the very first growth cycle was in medium containing both methanol and succinate. This allows the large number of doublings from single colonies to half-maximal density to occur in succinate over the first ~24 h of the transfer cycle before the cells experience a transient lag during the diauxic shift to growth on methanol [27]. All subsequent growth was in medium containing just methanol. As desired, this small change in protocol for the first growth cycle prevented the variation in growth between replicate populations (Figure 1). None of these populations’ final density ever dipped below 3 × 10^7^ mL^-1^.

### Population E2 lost fitness at standard densities following growth at low density

**Figure 2:**
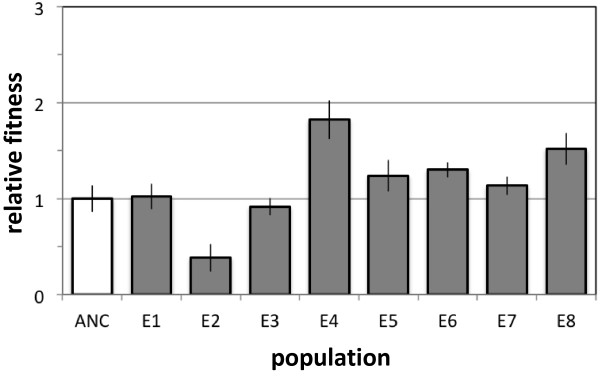
**Fitness of the E2 population tested at standard density had decreased by 84 generations.** Unlike the other seven populations which either improved or were indistinguishable from the ancestor, the E2 population fitness decreased by half when assayed against the ancestor with both competitors starting at the standard initial density (grey bars). ANC represents the control of the two color variants of the EM ancestor competed against each other (white bar). Data represent the mean and standard error.

Why, unlike the other five populations that only transiently dipped in cell density, did it take the did the E2 population over 100 generations to recover? The fitness of all populations was assayed via competitions at generation 84, which was before E2 had recovered to full density, but after all other populations had done so. In order to remove confounding effects of the final density of the acclimated culture for these fitness assays, aliquots of the frozen mixed populations were grown for the first cycle in methanol and succinate, as described above to initiate the ‘F’ populations. This allowed all populations, including E2, to achieve full cell densities before a second acclimation cycle with just methanol. For the competition assay, each strain was then inoculated into a single flask (1/128 of each; a net 1/64 dilution), and the ratio was determined at day zero and day four. Unlike all other populations that either improved or were indistinguishable from the ancestor by this point, the E2 population was approximately half as fit than its ancestor (Figure 2).

### Population E2 evolved to grow slower than its ancestor

**Figure 3:**
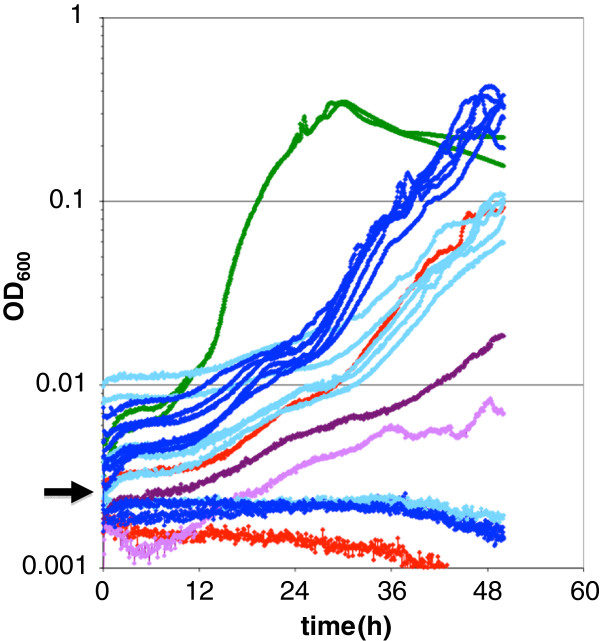
**Growth dynamics in 96-well plates indicate that the E2 population is slower than the ancestor.** Although much slower than wild-type (green), all populations other than E2 had increased their growth rate between generation 60 and 120 (light and dark blue). The E2 population, however, was slower than the EM ancestor (red) at both 60 and 120 generations (light and dark purple). Note that below a threshold OD_600_ of ~0.002, cultures other than E2 fail to recover, including one of the EM ancestors. Data represent the mean of three replicates on the plate.

In order to determine whether the pairwise fitness differences against EM observed above could be seen at the level of the growth of individual populations, growth of the ancestors and evolved populations at different timepoints were measured in a shaken 96-well plate in a plate reader (Figure 3). First, it was quite clear that growth of *M. extorquens* AM1 in a 96-well plate format resulted in unusual behavior and was far from the ideal method to assay growth. Compared to previous work in flasks [28], even wild-type displayed unexplained, yet repeatable growth dynamics typified by alternating periods of faster and slower rates of apparent growth, perhaps due to clumping. Most E populations showed similar growth dynamics to EM at generation 60, but were faster by generation 120. Notably, however, population E2 was slower than EM at both timepoints, consistent with the decreased fitness that had been observed. Additionally, as seen in the instance displayed here, some cultures appeared not to have fully acclimated prior to testing, as evidenced by the lower initial densities. Other than the E2 populations, none of these cultures with an initial reading below ~0.002 during this particular experiment ever began to grow during the measured duration; however, the same cultures grew without issue on other days when started above this density threshold (data not shown). For example, during this experiment one color variant of the EM ancestor grew normally whereas the other did not (Figure 3, in red).

### Density-dependence for growth was specific to methanol growth and the engineered ancestral genotype

The inability of populations to grow on a given day when started below a threshold inspired a direct examination of a possible threshold to initiate growth. Cultures were first grown to full density via a methanol plus succinate growth cycle followed by a transfer in just methanol. Starting from identical acclimation flasks to control for the physiology of the founding cells at time of transfer, cultures were inoculated into methanol medium at different starting densities. The EM ancestor grown in methanol recovered from dilutions up to 1/256 from a fully-grown culture (~1 × 10^8^ mL^-1^) in four days, but a 1/512 dilution from the same culture took ten days to establish the same final count and greater dilutions never recovered. Further experiments revealed that this density-dependence changed over the course of adaptation, was specific to the EM ancestor, and depended upon which growth substrate was utilized. First, after 180 generations the E2 population could recover from a 1/(2^11^) = 1/2,048 dilution in four days. Second, wild-type could recover in the same four days from a 1/(2^19^) = 1/524,288 dilution. This demonstrated that growth threshold is thus not a general feature of culturing *M. extorquens* AM1 in methanol, but is specific to EM. Finally, in succinate medium EM could recover from a 1/(2^12^)= 1/4,096 dilution in just two days, or a 1/(2^22^) = 1/4,194,304 (an inoculum of ~300 cells) in five days. This established that density-dependence was not simply a general feature of this strain, rather, it was specific to the combination of the changed formaldehyde oxidation pathway and growth in methanol medium in which this pathway is required.

### Population E2 specifically adapted to growth at low cell densities with tradeoffs at higher densities

**Figure 4:**
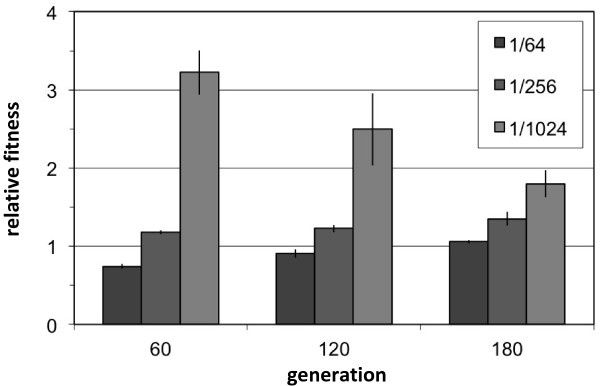
**Population E2 adapted to low density growth with tradeoffs at the standard high density.** Competitions against the EM ancestor were performed using the same set of acclimation cultures that were combined 1:1 and diluted to varying degrees (indicated by the shade of grey). Note E2 fitness at generation 60 is more than four-fold higher at the lowest versus the highest starting density, and that this low-density specificity waned as they evolved. Data represent the mean and standard error.

What might explain the early decrease in fitness of the E2 population? Although, the above competitions were initiated from cultures grown to ~1 × 10^8^ mL^-1^, E2 spent essentially all of its first 120 generations at densities at least an order of magnitude below this before finally recovering by generation 180. Might these low densities essentially represent a different ‘environment’ to the cells despite being identical in terms of the medium provided, thus resulting in a distinct physiological state? If so, it is possible that the E2 population that became less fit at standard, high densities did so as a consequence of adaptation that was specific to the low densities it actually experienced. In order to test for a possible density-dependence of the relative fitness of the E2 population vs. its EM ancestor, competition assays were performed from a single set of acclimation flasks – grown as above to full density and ensuring identical initial physiological states – that were then diluted to varying extents (Figure 4). This design maintained both the ratio and physiological state of competitors across treatments that differed only in the initial total cell number. Consistent with results from generation 84, the E2 population at generation 60 was less fit than EM when growth started following the standard 1/64 dilution. Remarkably, at just a four-fold greater dilution (1/256) the E2 population was more fit than EM, and at a further four-fold greater dilution (1/1024) it was more than three-fold more fit. This indicated that the E2 population had actually gained fitness at low densities, but that this improvement came at a strong tradeoff when grown at just 16-fold higher initial cell densities. By comparison, similar tests with performed with one of the other populations (E1 at generation 120) revealed fitness gains of 35-40% over the ancestor that were consistent over all densities tested.

### Low density-specific adaptation was lost as the E2 population recovered to full density

The E2 population improved dramatically in their ability to grow at low cell densities. By generation 180, however, they had recovered to the standard density seen for all of the other replicate populations. Given that their early fitness gains at low density came at a tremendous tradeoff to growth at higher density, might they have lost this capacity with time spent at high density? Competitions of the E2 population at 120 and 180 generation against the EM ancestor at various densities indicated that fitness at typical densities eventually surpassed that of EM, but that in turn the advantage at low densities waned substantially (Figure 4).

## Discussion

### Surprising discovery of a persistent, environment- and genotype-specific density threshold for growth

This work describes an unexpected density threshold for growth that was specific to the combination of environment and genotype used. Only the EM ancestor grown on methanol displayed this behavior, whereas either growth of EM in succinate or the wild-type strain in methanol permitted recovery from quite large dilution factors. This finding is analogous to the work on invertase in yeast, where it was the combination of solitary genotypes and sucrose as a resource that led to a dramatic density-dependence in the ability to initiate growth [7].

Whereas most density-dependent phenomena that have been studied have involved examination of a single physiological transition such as the lag from a growing to non-growing culture [9], the drop in final cell density reported here persisted over many growth cycles. Of the six cultures that experienced a crash in final density, it took over 60 generations for five of these to recover, and more than 120 generations for the E2 population. If one assumes that these dynamics were based solely upon changes in growth, per se, rather than mortality, the 100-fold drop in final cell densities between the end of generation 12 and 24 – between which there were two 1/64 dilutions – would translate into less than six doublings of these cultures in the intervening eight days (a net doubling time of ~36 h). On the other hand, bouncing back from this low point would have required ‘extra’ doublings beyond the 64-fold dilution to have climbed back up in number. Although unusual for the chronological duration it lasted (24 generations = 16 days), the synchronicity of the fall and rise of the six populations that transiently crashed is consistent with purely phenotypic acclimation independent of new mutations that would be expected to arise and escape drift at different times. On the other hand, as clearly occurred for population E2, it is possible that the return to high density involved some degree of evolutionary adaptation, with new genotype(s) rapidly taking over during the recovery.

### Adaptation to growth at low cell densities led to substantial tradeoffs at higher densities

There are two non-exclusive explanations for the decrease in performance of the E2 population at standard cell densities: fixation of deleterious alleles by drift or an inappropriate match between the actual selective environment and that in which the competitions were conducted. Addressing the first possibility, the cell densities to which E2 plummeted to resulted in a minimal bottleneck of ~1.5 × 10^4^ cells, which was much smaller than the typical bottleneck of 1.5 × 10^7^ when at the expected final density. This is far too large, however, to permit substantially-unfit genotypes to rise due to drift, and even then, the change occurred much too rapidly to be explained by a neutral process.

The evidence strongly argues that the cause of the unusual phenotypes reported here was adaptation specific to the physiology experienced by the E2 population at sustained low densities. The more than 4-fold swing in relative fitness across a 16-fold range in initial cell densities indicates that, not only did the E2 population improve at low density, there was antagonistic pleiotropy that generated tradeoffs between these ‘environments’. It is not unreasonable to speculate, given the density-dependent growth differences seen, that there will have been significant changes in global gene expression at various densities. As such, some mutations may have been advantageous only to those cultures which dipped to such low densities, and analogously might be neutral or deleterious at high density. The result at the lowest density tested, if anything, might be an underestimate of the actual initial advantage of this phenotype, as around generation 24 it was 400-fold below its cohorts that never dipped. Ironically, the success of the E2 population eventually led to it returning to standard cell densities and a decline in low-density performance.

Research into the underlying the physiological and genetic bases of adaptation to low density in the E2 population is ongoing. Possible mechanisms would either involve the production of a compound that promotes growth or removal of an inhibitory component. The association of the phenomenon with methanol, and not succinate, growth has parallels with a previous observation of cobalt limitation in this growth medium [29]. All F populations evolved to be able to grow well in low cobalt medium, six of these via transposition of a particular insertion sequence upstream of a novel cobalt transporter that increased its expression. The link between cobalt and methanol (vs. succinate) was shown do be due to the growth-dependent dilution of adenosylcobalamin needed for enzymes of a pathway specifically required for growth on C_1_ compounds. What distinguishes the density-dependent phenomenon described here from prior results, and thus argues against these results being due to the same limitation, was the differential behavior of EM and wild-type. Cobalt limitation was strongest at high growth rates, such that it depressed wild-type growth much more than that of the slow EM ancestor [29]. Here the opposite was observed: wild-type recovered from a cell inoculum 1000-fold lower than EM. Perhaps the density-dependence is not even so much unique to the EM ancestor, per se, but is more an indirect effect if their slow growth, such that even general processes such as CO_2_ production occur more slowly than in wild-type. Given there is precedence for CO_2_ stimulation (or inhibition) of growth [10], and the fact that the serine cycle required for growth on methanol (but not succinate) is partially autotrophic, this is at least a plausible scenario. Ultimately, uncovering the answer will likely require our current efforts to re-sequence the genome of an E2 isolate to determine the genetic basis.

## Conclusions

### A cautionary tale for starting to evolve experimental populations

One implication of this finding is inherently quite pragmatic: be careful about the details of starting an evolution experiment! In this case, taking care to avoid one complicating factor – the use of a single starter batch that can lead to genetic parallelism due to repeated selection of rare, fit variants – led to a different problem. Because some populations did not achieve the expected final density from the small inoculum of a colony, they started to fall further and further behind with each of the first four transfers. This entire phenomenon was very easily avoided with the F populations that followed by simply including a rapidly utilizable substrate (succinate) in addition to methanol to reliably bring populations up to near-maximum density from the start. The massive tradeoffs that accompanied the adaptation of the E2 populations to growth at low density were quite interesting on their own, but complicated the original goals of the project enough that a restart was deemed necessary.

### Relevance for considering evolution of natural populations that exist at low density?

Microbes isolated from natural environments where they grow to very low densities tend to have properties that preclude them from being successful at higher densities. As this has been increasingly recognized, new strategies have emerged to try to obtain in pure culture those phyla that had previously escaped cultivation [13,14]. More recently, approaches that seek to use co-culturing or molecules that might influence each other have been shown to also expand the range of microbes brought into culture [30]. What is lacking, however, are examples of transitions between life at different densities. This model system or others with similar properties offer the unique opportunity to uncover physiological mechanisms that underlie both adaptation to different cell densities and the tradeoffs that can emerge as a consequence.

## Methods

### Bacterial strains and growth conditions

Two different engineered *M. extorquens* AM1 (EM) strains were used as population ancsetors in this study, CM701 or CM702 [25]. These strains contain deletions of the first dedicated enzyme in H_4_MPT biosynthesis, β-ribofuranosylaminobenzene 5′-phosphate synthase, encoded by *mptG*[31]. CM701 is the Δ*mptG* strain CM508 [32] with the pCM410 plasmid expressing *flhA* and *fghA*[25], whereas CM702 is the Δ*mptGcrtI*^*502*^ strain CM624 [32] also containing pCM410. Comparisons to the wild-type *M. extorquens* AM1 used the strains CM501 or the *crtI*^*502*^ strain CM502 [32]. The *crtI*^*502*^ allele encodes a disrupted phytoene desaturase, and thus eliminates synthesis of the pink carotenoids typically found in *Methylobacterium*[33], and has served as a neutral marker to monitor for contamination or for fitness assays [34].

All strains were cultured at 30 °C in “Hypho” minimal medium containing Vishniac trace metal solution made as described previously [34], using either 15 mM methanol or 3.5 mM disodium succinate as carbon sources for liquid growth, unless otherwise noted. For the EM strains this results in ~1 x 10^8^ cells mL. The evolving populations and most subsequent analyses occurred in a volume of 9.6 mL liquid medium in 50 mL flasks that were shaken at 225 rpm.

Growth analyses in 96-well plates occurred in 200 μL of medium per well, and were both cultured with constant orbital shaking and had changes in OD_600_ measured every 5 minutes within a VersaMax plate reader (Molecular Devices). The data displayed are the average of three replicates. Variation was fairly low; for example, there was 4.4% average variation between replicates throughout exponential growth of wild-type.

Populations E1-E8 were grown on methanol minimal medium and were each established directly from single colonies of CM701 or CM702, respectively. The four odd numbered ‘E’ populations were established with the pink CM701 strain, whereas the even populations began with the white CM702 strain. Every 4 days 150 μL was transferred into 9.45 mL of fresh medium, a 1/64 dilution that permitted an average of six generations of growth per cycle and a final population size of ~1x10^9^. Population samples were cryo-preserved in 8% v/v DMSO and stored at -80 °C. For the first 120 generations this occurred every other transfer (12 generations), and then every five transfers (30 generations) from that point until 210 generations. In order to assess possible contamination and obtain estimates of final growth densities, following the transfer to new medium dilutions were plated onto solid medium containing agar (1.8% w/v, Difco) supplemented with 125 mM methanol to permit larger colonies to form. At generation 36, zero colonies were observed for populations E2 and E8; a value of one colony was used to plot their approximate density, but these represent just estimates.

Subsequent to the propagation of the eight E populations, new populations of the same two EM strains were established in the same manner with one key difference. These ‘F’ populations described previously [25,29] were started from individual colonies but were allowed to grow in a mixture of methanol and succinate (7.5 mM and 1.75 mM, respectively) for the first transfer. This allowed sufficient cell density to be obtained to avoid the ill effects of low density observed for the E populations.

### Fitness assays

The relative fitness of evolved populations was determined as before [34] using carotenoid biosynthesis as a visible, neutral marker. Although fluorescence and flow cytometry is now commonly used for this purpose due to improved precision, this method had not been developed at the time of these experiments and both methodologies have been demonstrated to produce similar results [34]. Relative fitness (*W*) of the mixed populations compared to the reference strain (EM ancestor of the opposite color) was calculated by a previously described equation for mixed populations during competitive growth:

$$W_{\mathrm{ij}}=\log\left(\frac{N_{i1}}{N_{i0}}\right)/\log\left(\frac{N_{i1}}{N_{i0}}\right)$$

where *N* is the number of cells in the competition (accounting for different dilution factors used for initial and final counts, particularly experiments started at low density) of strain *i* or strain *j* at beginning (*0*) or ending (*1*) of the growth cycle [35]. Data are presented as the mean and standard error of at least three replicates.

Fitness assays utilized cultures that were first acclimated from the freezer for four days in medium containing both methanol and succinate at half their usual concentration (7.5 mM and 1.75 mM, respectively) in order to allow sufficient growth from the frozen stock. A standard transfer of 1/64 of this first culture was used to start a second four-day period of growth in methanol alone (15 mM). In general, competitions were established with 1/128 dilutions of each competitor, mixed by vortexing, and an initial timepoint was sampled and diluted appropriately for plating prior to placing the flasks back into the incubator. Competitions were allowed to proceed for four days and then a sample was diluted and plated to obtain final counts of each competitor. In order to test the effect of absolute density upon fitness, smaller amounts of the same competitors were inoculated into new medium, resulting in either 4- or 16-fold lower initial cell densities (but at the same relative frequencies). This 16-fold range of experimental treatments overwhelmed a 67% range in starting densities for competitions due to differential final densities in the acclimation cultures.

## Competing interests

The author declares no competing interests.

## Authors’ contributions

CJM performed and analyzed the experiments, was responsible for the overall design and direction of the experiments, wrote the paper, and approved the final manuscript.
